# Flow Density in Optical Coherence Tomography Angiography is Useful for Retinopathy Diagnosis in Diabetic Patients

**DOI:** 10.1038/s41598-019-45194-z

**Published:** 2019-06-17

**Authors:** Yoshihiro Kaizu, Shintaro Nakao, Mitsuru Arima, Takehito Hayami, Iori Wada, Muneo Yamaguchi, Haruka Sekiryu, Keijiro Ishikawa, Yasuhiro Ikeda, Koh-Hei Sonoda

**Affiliations:** 1 Department of Ophthalmology, Graduate School of Medical Sciences, Kyushu University, Fukuoka, Japan; 2 Department of Intelligent Mechanical Systems, Graduate School of Natural Science and Technology, Okayama University, Okayama, Japan

**Keywords:** Predictive markers, Eye diseases

## Abstract

Our study evaluated the diagnostic capability of flow density (FD) in OCT angiography (OCTA) for diabetic retinopathy (DR) detection in diabetic patients. We studied 93 eyes of 68 diabetic patients who underwent OCTA (36 and 57 eyes without and with DR, respectively). Retinal capillary FD of a 2.6 × 2.6 mm^2^ area and four divided areas at the superficial (SCP) and deep capillary plexus (DCP) were measured. Predictions were evaluated using the area under the receiver operating characteristic curve (AUC). The diagnostic capabilities of the FDs in discriminating between eyes without DR and eyes with total or early DR were compared. Furthermore, predictions with foveal avascular zone (FAZ) area, hemoglobin A1c (HbA1c), and DM duration were also compared with FD. Prediction using FD AUC in the temporal side in the DCP (0.83) was the highest and significantly better than all other AUCs examined (*P* < 0.05), including discriminating between eyes without DR and with early DR (*P* < 0.01). Prediction using this particular AUC was also significantly better than that by FAZ area and HbA1c (*P* < 0.001 and <0.001, respectively). Area-divided FD in OCTA may be valuable for diagnosing retinopathy in diabetic patients.

## Introduction

Without diabetic macular edema (DME) or vitreous hemorrhage, diabetes mellitus (DM) patients rarely experience subjective symptoms regarding their visual acuity in the early and even middle stages of diabetic retinopathy (DR). Early diagnosis for treatment such as glycemic control can prevent DR progression and subsequent declining visual function^1–3^. It is therefore important to detect DR as early as possible. It has been reported that fundus observation using fundus photographs or mydriatic ophthalmoscope^4,5^, electroretinography^6^, DM duration^7,8^, and hemoglobin A1c (HbA1c)^9^ are useful parameters for detecting or predicting DR.

DR shows capillary abnormality following hyperglycemia or abnormal blood sugar change, and capillary obstruction and subsequent retinal ischemia are important in its pathogenesis^10^. Various studies have observed localization of microaneurysms in the border zone of acellular capillaries, suggesting that retinal ischemia precedes the microaneurysms^11^. However, there is limitation in existing examination techniques for effective evaluation of capillary dropout, and therefore microaneurysms, intraretinal bleeding, venous dilatation or abnormal blood vessels detectable by fundus examination have been used for DR diagnosis^12^.

Optical coherence tomography angiography (OCTA) can noninvasively image the retinal vessels at the capillary level^13,14^. Furthermore, retinal capillary flow density (FD) quantification is possible using OCTA^15^, and FD evaluation is a clinically adequate method for gross assessment of DR severity^16–18^. Furthermore, we observed that capillary dropout is dominant in the deep capillary plexus in early diabetic retinopathy in OCTA^19^. However, it is unknown if FD in OCTA is useful for DR diagnosis. In this study, we investigated the diagnostic ability of FD in OCTA to detect DR among eyes from DM patients.

## Results

A total of 93 eyes of 68 patients (21 female) were evaluated in this study (60 right eyes and 33 left eyes). Thirty-six eyes had no diabetic retinopathy (NDR) while 57 eyes did, including 40 with early DR (15 with mild nonproliferative diabetic retinopathy (NPDR) and 25 with moderate NPDR) and 17 with late DR (six with severe NPDR and 11 with proliferative diabetic retinopathy (PDR)). Detailed patient characteristics are shown in Table 1. There was no statistical difference in age, HbA1c and axial length between the two groups (*P* = 0.44, *P* = 0.27, *P* = 0.28, respectively). However, DM duration of the DR group was significantly longer than that of the group without DR (*P* = 0.01). One and five patients with type 1 DM were included in the NDR group and DR group, respectively.

**Table 1:** Characteristics of All Study Subjects.

Characteristics	NDR group	Mild + Moderate NPDR group	All DR group	*P*
Sex (male/female)	16/9	20/11	31/12	—
Age (years)	58.3 ± 16.0	55.1 ± 14.8	55.4 ± 14.1	0.44
Type of DM (Type1/Type2)	1/24	5/31	5/38	—
Duration of DM (years)	7.0 ± 6.2	7.6 ± 1.1	12.7 ± 9.6	0.01
HbA1c (%)	7.4 ± 1.6	7.8 ± 1.3	7.8 ± 1.3	0.27
Axial length (mm)	24.2 ± 1.2	23.9 ± 1.0	23.8 ± 1.1	0.28

### Retinal Flow Density of Subjects

**Figure 1:**
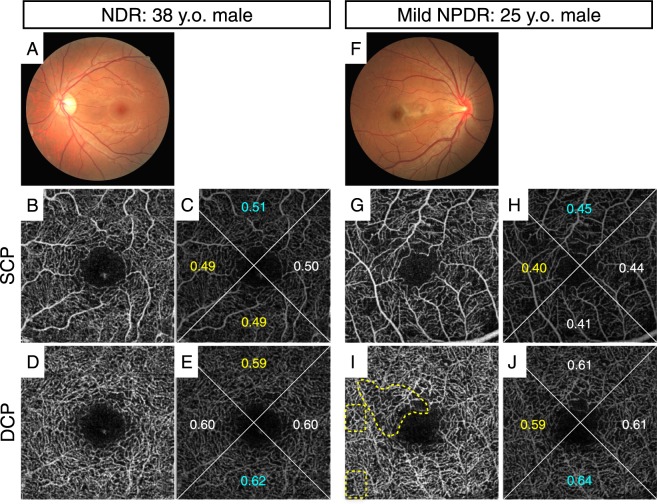
Representative cases of an eye without DR (NDR, 38 y.o. male, HbA1c 6.5%, DM duration 2 years; (**A**–**E**) and an eye with DR (mild NPDR, 25 y.o. male, HbA1c 7.7%, DM duration 1 year; (**F**–**J**). This NPDR case showed local capillary dropout and higher orientation bias compared with the NDR case although whole flow density (FD) in NPDR was not decreased as compared with that of NDR. (**A**,**F**): color fundus photograph, (**B**,**C**,**G**,**H**): 2.6 × 2.6 mm SCP images, (**D**,**E**,**I**,**J**): 2.6 × 2.6 mm DCP images. Numbers indicate FD of each area in (**C**,**E**,**H**,**J**). The FD of the whole area in (**C**,**E**,**H**,**J**) was 0.50, 0.60, 0.43, and 0.61, respectively. Yellow dotted circles indicate capillary dropout (**I**).

The FAZ area (mean ± SD) of the NDR, mild and moderate NPDR and DR groups were 0.34 ± 0.12 mm^2^, 0.35 ± 0.10 mm^2^ and 0.42 ± 0.18 mm^2^, respectively. While there was no significant difference in FAZ area between the NDR group and the mild and moderate NPDR group (*P* = 0.96), the FAZ area of the DR group were significantly larger than that of the NDR group and the mild and moderate NPDR group (*P* = 0.02 and 0.04, respectively). FD values in all regions of the superficial capillary plexus layer (SCP) and deep capillary plexus layer (DCP), and the orientation bias ratio of FD in the SCP and DCP of the DR group were significantly smaller than those of the NDR group. However, FD values in all regions and the orientation bias ratio of FD in the mild and moderate NPDR group were significantly smaller than those of the NDR group in DCP but not in SCP (Table 2). The FD of each area and the orientation bias ratio of the NDR, mild and moderate NPDR, and all DR groups are respectively shown in Table 2. Figure 1 shows representative cases of NDR and mild NPDR. The NPDR case showed local capillary dropout and higher orientation bias compared with the NDR case although whole FD in NPDR was not decreased as compared with that of NDR.

**Table 2:** Values of Flow Density of Each Region Without Foveal Avascular Zone and the Orientation Bias Ratio in NDR Group, mild + moderate NPDR Group, and All DR Group (*n* = 36, 39, and 57, respectively).

	NDR group	Mild + Moderate NPDR group	All DR group
**Superficial Capillary Plexus Layer (SCP)**			
Superior	0.45 ± 0.10	0.43 ± 0.08	0.39 ± 0.09**
Inferior	0.45 ± 0.09	0.42 ± 0.09	0.39 ± 0.09**
Temporal	0.45 ± 0.08	0.41 ± 0.08	0.38 ± 0.08**
Nasal	0.45 ± 0.08	0.43 ± 0.08	0.39 ± 0.09**
Whole (2.6 × 2.6 mm)	0.45 ± 0.09	0.42 ± 0.08	0.39 ± 0.09**
Orientation bias ratio	0.92 ± 0.05	0.90 ± 0.06	0.88 ± 0.07*
**Deep Capillary Plexus Layer (DCP)**			
Superior	0.57 ± 0.11	0.49 ± 0.11**	0.45 ± 0.12***
Inferior	0.58 ± 0.11	0.48 ± 0.12**	0.44 ± 0.13***
Temporal	0.57 ± 0.10	0.47 ± 0.10**	0.43 ± 0.11***
Nasal	0.57 ± 0.10	0.49 ± 0.10**	0.44 ± 0.12***
Whole (2.6 × 2.6 mm)	0.57 ± 0.10	0.49 ± 0.10**	0.44 ± 0.12***
Orientation bias ratio	0.93 ± 0.05	0.89 ± 0.07*	0.88 ± 0.08**

The significance of the differences was analyzed using the Tukey–Kramer test.

*, **, and *** Symbols depict a statistically significant difference compared to the NDR group with *P* value < 0.05, <0.01, and <0.001 respectively.

### Area Under the Curve

**Figure 2:**
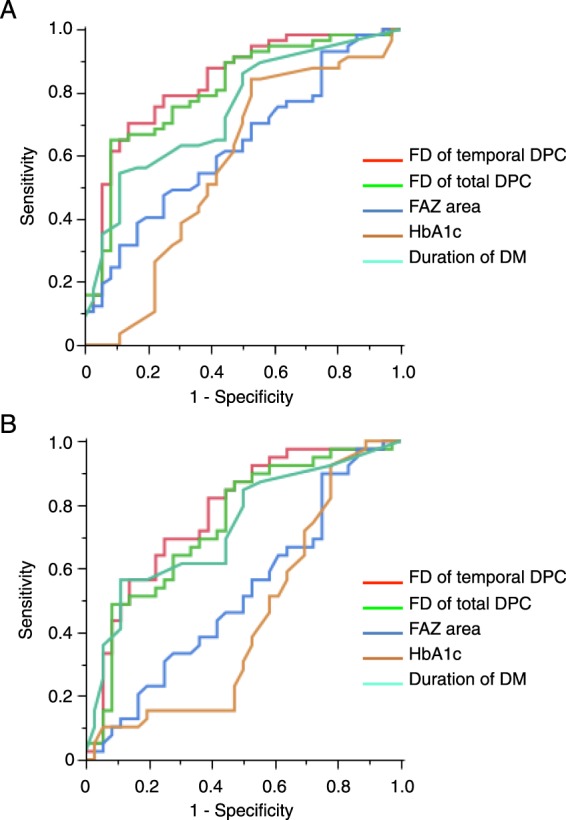
Receiver operating characteristic (ROC) curves and area under ROC curve (AUC) of the temporal DPC flow density (FD) (red), the total DPC FD (green), FAZ area (dark blue), HbA1c (brown), and DM duration (light blue) with respect to distinguishing between NDR and eyes with any stage of DR (**A**) or between NDR and eyes with early DR stage (i.e. mild or moderate nonproliferative diabetic retinopathy)(**B**). (**A**) FD of the temporal side in the DCP has a significantly higher AUC than the other parameters except for DM duration. (**B**) FD of the temporal side in the DCP has a significantly higher AUC than the FD of the total DCP.

Next, based on FD, we calculated the area under the ROC curve (AUC) of each region and the orientation bias ratio of FD to evaluate the DR detection ability (Table 3). The region with the greatest AUC was the temporal side in the DCP (0.83; 95% CI = 0.73–0.90). The AUC of the temporal side in the DCP was higher than that of the other nine regions and the orientation bias ratio in the SCP and DCP (*P* < 0.05). Thereafter, we compared the ability of FD of the temporal side in the DCP with other parameters such as FAZ area, HbA1c, and DM duration. The FD of the temporal side in the DCP had significantly higher AUC than FAZ area and HbA1c but not DM duration (*P* < 0.001, <0.01 and 0.08 respectively) (Table 4, Fig. 2A). Furthermore, we also calculated the AUC of each region and the orientation bias ratio of the FD to discriminate between NDR and eyes with mild or moderate NPDR. The FD of the temporal side in the DCP also had significantly higher AUC than the FD of the whole area in the DCP (*P* < 0.01 respectively) (Table 5, Fig. 2B).

**Table 3:** Area Under the ROC Curve (AUC) of FD in Each Region and Orientation Bias Ratio.

	AUC	95% CI
**Superficial Capillary Plexus Layer (SCP)**		
Superior	0.67	0.55–0.77
Inferior	0.69	0.56–0.79
Temporal	0.70	0.58–0.80
Nasal	0.67	0.55–0.77
Whole (2.6 × 2.6 mm)	0.69	0.57–0.79
Orientation bias ratio	0.69	0.56–0.79
**Deep Capillary Plexus Layer (DCP)**		
Superior	0.79	0.67–0.87
Inferior	0.79	0.68–0.87
Temporal	0.83	0.73–0.90
Nasal	0.81	0.70–0.88
Whole (2.6 × 2.6 mm)	0.81	0.70–0.88
Orientation bias ratio	0.73	0.61–0.82

**Table 4:** Area Under the ROC Curve (AUC) of FD of the Temporal Side in the DCP and Other Parameters.

	AUC	95% CI	*P* Value
FD of temporal side in Deep Capillary Plexus Layer (DCP)	0.83	0.73–0.90	—
FD of whole area in DCP	0.81	0.70–0.88	0.017
Foveal avascular zone area	0.63	0.51–0.74	<0.001
Hemoglobin A1c	0.57	0.44–0.70	<0.01
Duration of DM	0.74	0.63–0.83	0.08

Each *P* value represents significance in comparison between FD of the temporal side in the DCP and other parameters regarding discrimination between NDR and eyes with any stage of DR.

**Table 5:** Area Under the ROC Curve (AUC) of FD of the Temporal Side in the DCP and Other Parameters.

	AUC	95% CI	*P* Value
FD of temporal side in Deep Capillary Plexus Layer (DCP)	0.77	0.65–0.86	—
FD of whole area in DCP	0.74	0.61–0.84	<0.01
Foveal avascular zone area	0.52	0.39–0.65	<0.001
Hemoglobin A1c	0.44	0.31–0.58	<0.001
Duration of DM	0.73	0.60–0.83	0.44

Each *P* value represents significance in comparison between FD of the temporal side in the DCP and other parameters regarding discrimination between NDR and eyes with mild or moderate NPDR.

## Discussion

Fundus photographs and fundus examination have been the gold standard for DR screening^4^. Microaneurysm detection is based on current international severity classification based on fundus findings^12^, and a previous pathology could indicate the presence of microaneurysms around the capillary dropout in DR^11^. It is possible that ischemia-induced VEGF generates microaneurysms^20–22^. Furthermore, it has been reported that OCTA cannot image all microaneurysms^23^. These reports motivated us to hypothesize that capillary dropout detection is fundamental to early DR detection. Our study shows that the FD by OCTA representing vascular nonperfusion at the capillary level is useful for diagnosis.

Various studies with OCTA have confirmed enlarged FAZ in eyes with DR^24^. However, large interindividual variability of FAZ has also been reported^25^. Our study analysis showed that the AUC of FAZ was relatively low. This indicates FAZ measurement might be unsuitable for DR diagnosis despite its significant correlation with visual acuity^26^.

Previous pathology and imaging studies have reported that vascular abnormalities occur more frequently in the temporal area than in the nasal area^27,28^. We also confirmed this spatial bias of DR-related vascular damage at the capillary level with OCTA and it could only be detected in the DCP in DR^18^. Interestingly, our results found a significant difference in the ability of different areas to detect DR and early DR as measured by AUC. Furthermore, our data showed that region-specific measurement of FD is more useful for DR diagnosis than FD of the whole retina.

Various OCTA quantitative data have also demonstrated that the FD in some NDR cases was higher than in some NPDR cases as shown in Figure 1, although the average NDR FD was significantly lower than the average NPDR FD^16–18^. We recently reported that DR-related capillary dropout arises locally and randomly with OCTA^18^. The quantitative analysis showed spatial bias of FD in DR eyes^18^. The clinical relevance of the spatial pattern of capillary dropout in DR has not yet been determined. In this study, however, the AUC of spatial bias was not as high as that of region-specific FD. Even in early DR, there may be cases where capillary dropout occurs across several areas.

Although color fundus photographs are widely used for detection of DR^4^, there are several disadvantages to it as compared with OCTA. First, color fundus photographs and ocular examination require pupil dilation which temporarily worsens the patient’s vision while OCTA does not. Second, the quality of color fundus photographs is affected by the presence of cataract while OCTA is less affected as OCTA utilises a long laser light. Third, ocular examination is a subjective method and thus exhibits inter-rater variability, whereas OCTA is objective and the data from OCTA is highly reproducible even in DR patients^29^. These factors further contribute to the usefulness of OCTA for detection of DR.

This study has the limitations inherent in any study of limited sample size. Another limitation is the exclusion of patients with macular edema or vitreous hemorrhage although these patients are representative of DR pathogenesis. Furthermore, this study included both eyes from some participants while only one eye from some patients. This might affect the accuracy of this study. An additional limitation is the small field of view, although vascular abnormalities frequently occur in the periphery of DR.

OCTA can noninvasively and quantitatively measure FD of the retinal vessels at high reproducibility without mydriasis^30^. Therefore, our current data suggests that FD quantitation in OCTA may be useful to detect DR in DM patients. Future investigation in a larger cohort is necessary to validate our results.

## Methods

This study was approved by the Institutional Ethics Committees of the Kyushu University Hospital (28-473, UMIN 000028656), and was performed in accordance with the ethical standards laid down by the Declaration of Helsinki. Written informed consent for the research and publication of this study and any accompanying images was obtained from all participants.

### Participants

This retrospective, observational and cross-sectional study included 93 eyes of 68 consecutive patients with type 1 or type 2 DM. All patients underwent OCTA at Kyushu University Hospital between November 2014 and November 2017. Exclusion criteria included the presence of significant DME. Furthermore, we also excluded eyes with poor quality OCTA images due to cataract, vitreous hemorrhage or poor fixation. In this study, all fundus examinations were performed by two retina specialists (SN, MA).

### Optical coherence tomography angiography

We obtained all OCTA images (superficial and deep) using the RTVue XR Avanti (Optovue Inc, Fremont, California, USA). This instrument has an A-scan rate of 70, 000 scans per second, using a scan light centered at 840 nm with a bandwidth of 45 nm. The tissue resolution is 5 µm axially. Each B-scan contained 216 A-scans. Five consecutive B-scans (M-B frames) were captured at a fixed position before proceeding to the next sampling location. The scanning areas were a 3 × 3 mm cube centered on the fovea, and we obtained retinal microvascular map images of these areas using OCTA. For each scan, superficial and deep layer OCTA images were generated based on the full automatic retinal segmentation performed by the OCT device software. The definitions of the segmentation are as follows. The SCP was defined by the top layer being the inner limiting membrane (ILM) with a 3 micron offset, and the bottom layer being the inner plexiform layer (IPL) with a 15 micron offset. The DCP was defined by the top layer being the IPL with an offset of 15 microns and the bottom layer being the IPL with an offset of 70 microns. Moreover, each en face 3 × 3 mm OCTA image (SCP and DCP) was cropped to a 2.6 × 2.6 mm square image centered on the fovea, because we predicted that the “angio FLOW” marks displayed on lower left of the 3 × 3 mm en face images would affect the quantitative results of the FD.

### Foveal avascular zone measurement

FAZ area was defined as the inside area of the inner boundary of the central capillary ring in the en face SCP image. Each FAZ area was manually traced using Image J software (version 1.51f; http://imagej.nih.gov/ij/; provided in the public domain by the National Institutes of Health, Bethesda, MD, USA) by a single grader. Then, using commercial software (NI Vision; National Instruments Corp., Austin, TX, USA), we calculated these outlined areas in pixels and these were converted into square millimeter based on the 606 pixels width of the original 3 × 3 mm images.

### Flow density measurement

For the aforementioned reasons, all 3 × 3 mm en face OCTA images were cropped to 2.6 × 2.6 mm using an image editing program (Photoshop; Adobe Systems, Inc., San Jose, CA, USA) (Supplemental Fig. 1A,B). Image binarization was then performed using NI Vision, and each image was uniformly divided into four sections (superior, inferior, nasal and temporal sides) (Supplemental Fig. 1C,D). The FD of each area was quantified without FAZ using NI Vision (Supplemental Fig. 1C,D). We defined FD as the proportion of pixels occupied by capillaries with blood flow compared with each total area.

### Predictive factors of diabetic retinopathy

We obtained participant demographic characteristics that may be predictive factors of DR, including HbA1c (%) and DM duration (years). Furthermore, we also calculated the orientation bias ratio (minimum FD/maximum FD) for each case and each layer (SCP and DCP) from the maximum and minimum FD values of the image in four directions as a predictive factor of DR. These parameters were compared for their DR detection ability with that of the FAZ area and the regional FD (whole 2.6 × 2.6 mm, superior, inferior, nasal and temporal).

### Comparison of diagnostic ability of diabetic retinopathy

To evaluate and compare the DR diagnostic ability, a receiver operating characteristic (ROC) curve of each parameter was constructed and the AUC was calculated. AUC is the statistical parameter that represents the disease diagnostic ability, and ranges from 0.5 (very poor) to 1 (excellent).

### Statistical analysis

All data were expressed as mean (SD). Statistical analyses were performed using the software, JMP® Pro 12.2.0 (SAS Institute, Cary, NC). Data that were not normally distributed were analyzed by nonparametric statistics. The significance of the differences was analyzed by the Tukey–Kramer test. *P* value < 0.05 was considered statistically significant.

### Ethics approval

The Institutional Ethics Committees of the Kyushu University Hospital (28-473, UMIN 000028656).

## Supplementary information

Supplemental Figure 1
