# Supplementary material for: Flow Density in Optical Coherence Tomography Angiography is Useful for Retinopathy Diagnosis in Diabetic Patients
Source: Sci Rep. 2019 Jun 17;9:8668. doi: 10.1038/s41598-019-45194-z (PMC6572797; doi:10.1038/s41598-019-45194-z)
Supplement: Supplementary file 1 — Supplemental Figure 1 [file 41598_2019_45194_MOESM1_ESM.pdf]

Flow Density in Optical Coherence Tomography Angiography is Useful for Retinopathy Diagnosis in Diabetic Patients

Yoshihiro Kaizu,[1] Shintaro Nakao,[1] Mitsuru Arima,[1] Takehito Hayami,[2] Iori Wada,[1] Muneo Yamaguchi,[1] Haruka Sekiryu,[1] Keijiro Ishikawa,[1] Yasuhiro Ikeda,[1] Koh-Hei Sonoda[1]

Address: [1]Department of Ophthalmology, Graduate School of Medical Sciences, Kyushu University, Fukuoka, Japan.

[2]Department of Intelligent Mechanical Systems, Graduate School of Natural Science and Technology, Okayama University, Okayama, Japan

Short Title: Flow Density in OCTA for DR diagnosis

Correspondence:
Shintaro Nakao, MD, PhD
Kyushu University
3-1-1 Maidashi, Higashi-Ku, Fukuoka 812-8582, Japan.
Tel: 81-92-642-5648
Fax: 81-92-642-5663
Email: snakao@med.kyushu-u.ac.jp

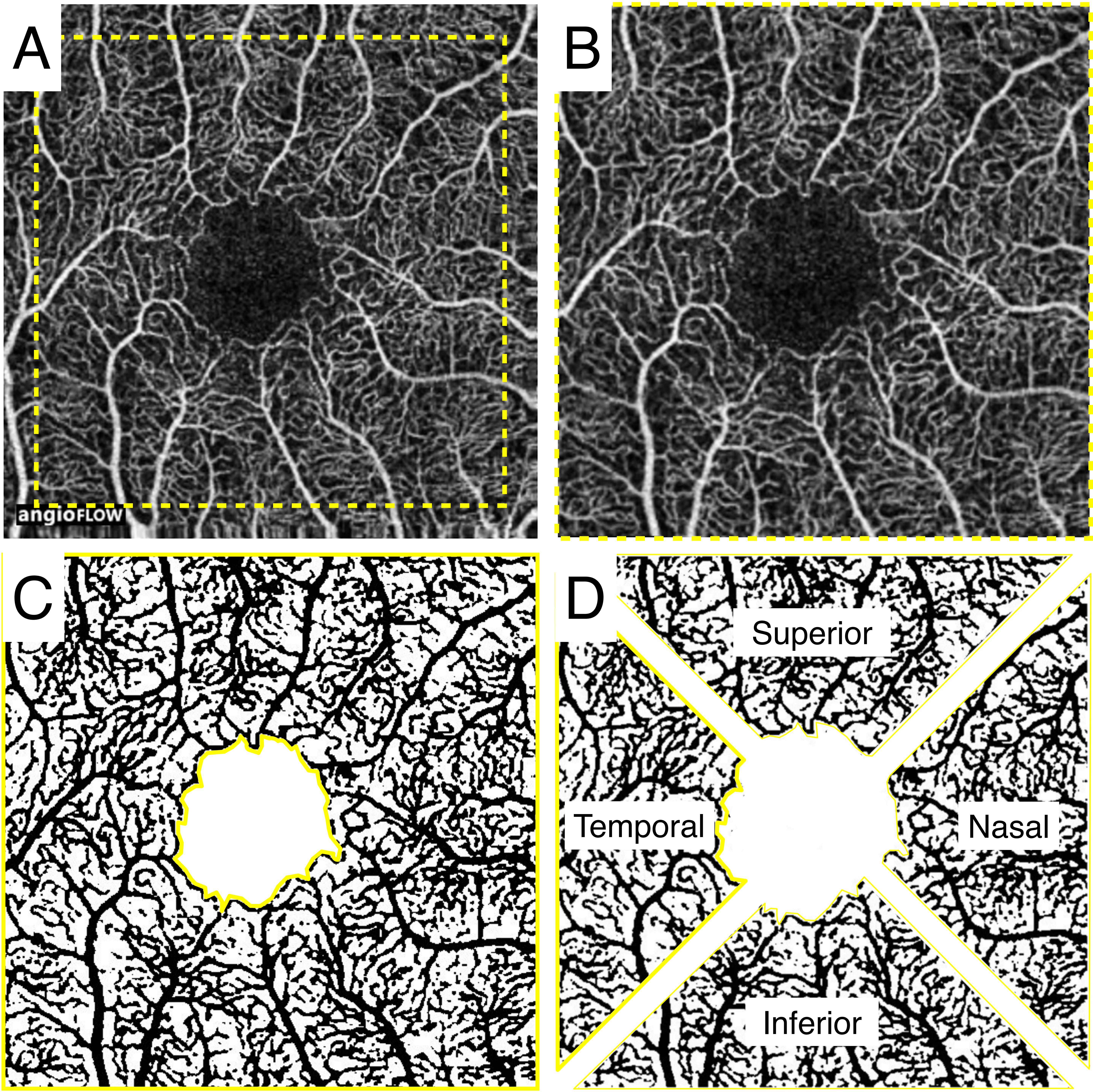

Supplemental Fig. 1. Quantification of flow density in OCTA. The OCTA image of a 45-year-old man at the SCP. (A) A 3 X 3 mm en face OCTA was cropped to 2.6 X 2.6 mm using an image editing program (Adobe Systems, Inc.; yellow dotted square) to avoid the influence of the “angioFLOW” display on quantification. (B) A 2.6 X 2.6 mm en face OCTA image used for this study. (C) Image binarization was performed using NI Vision. (D) Each image was uniformly divided into four regions (superior, inferior, nasal and temporal sides).
